# Dual-key slot mmwave antenna array with dual-band performance at 28 GHz and 38 GHz using characteristic mode analysis

**DOI:** 10.1038/s41598-025-33578-3

**Published:** 2026-01-19

**Authors:** Saad Hassan Kiani, Mohd Imran Ibrahim, B. G. Parveez Shariff, Muhammad Abbas Khan, Altaf Ahmed Mugheri, Tanweer Ali, Umair Rafique, Hala Mostafa, Mariana Dalarsson

**Affiliations:** 1 Faculty of Electrical and Electronic Engineering Technology, Universiti Teknikal Malaysia Melaka, Durin Tunggal, 76100 Malaysia; 2 Department of Advanced Communication Technology, Nitte University, NMAM Institute of Technology (NMAMIT), Nitte, 574110 India; 3 Department of Electrical Engineering, Balochistan University of Information Technology, Engineering and Management Sciences, Quetta, 83700 Pakistan; 4 Department of Electronics and Communication Engineering, Manipal Institute of Technology, Manipal Academy of Higher Education, Manipal, 576104 India; 5 Centre for Wireless Communicaitons, University of Oulu, Oulu, 90570 Finland; 6 Department of Information Technology, College of Computer and Information Sciences, Princess Nourah bint Abdulrahman University, P.O. Box 84428, Riyadh, 11671 Saudi Arabia; 7 School of Electrical Engineering and Computer Science, KTH Royal Institute of Technology, 100 − 44 Stockholm, Sweden

**Keywords:** Dual-band, Dual-key slot. MmWave, 28/38 ghz, Characteristics mode theory, Feed array, Gain, Engineering, Materials science

## Abstract

In this work, a dual key-shaped antenna for 28/38 GHz mmWave applications is designed, analyzed, and tested using the Theory of Characteristic Modes. The antenna is printed on a 0.254 mm thick substrate and has a compact footprint of 10 × 12 mm², making it suitable for modern space-limited 5G devices. It supports two operating bands centered at 28 GHz and 38 GHz, with measured fractional bandwidths of 12.5% and 21.05%, respectively. At the lower band, the first four characteristic modes dominate the radiation, while Modes 2, 3, 4, 6, and 10 mainly shape the higher-band performance. To further enhance gain and cover wider practical needs, the design is extended into a four-element linear array with an overall size of 19.75 × 26 × 0.254 mm³. The array provides peak gains of 10.5 dBi at 28 GHz and 11 dBi at 38 GHz, while maintaining more than 75% efficiency across both operating bands. A prototype of the antenna and array was fabricated and tested using in-house measurement facilities, showing good agreement with the simulated results. The proposed antenna system meets the key performance requirements for 5G mmWave communication and offers a compact and efficient solution for future 28/38 GHz wireless devices.

## Introduction

 The rapid growth in mobile data demand, driven by an increase in registered users and their need for high-quality multimedia content, has led to significant advances in mobile communication networks^1,2^. Fifth-generation (5G) wireless technology has emerged as a solution, promising ultra-fast data rates ranging from 5 to 50 Gbps. While earlier generations, including 2G, 3G, 4G, and LTE-Advanced, have primarily operated within the crowded sub-3.6 GHz spectrum, 5G aims to utilize higher frequency bands, especially in the millimeter-wave (mmWave) range, to satisfy the growing data requirements^3,4^. The mmWave spectrum, typically covering frequencies above 24 GHz, offers vast bandwidth potential for high-speed communication^5^. Notably, the 28 GHz and 38 GHz bands have garnered significant attention due to their favorable propagation characteristics for 5G applications^6–8^. Unlike lower frequency bands, mmWave frequencies can provide higher data rates, reduced latency, and increased network capacity, making them ideal for applications such as ultra-reliable low-latency communication, enhanced mobile broadband, and massive machine-type communication^9^. However, the adoption of mmWave technology for mobile communications comes with challenges, including higher free-space path loss, susceptibility to atmospheric absorption, and limited penetration through obstacles. Despite these challenges, the advances in antenna technology, such as beamforming and massive MIMO, are facilitating the effective deployment of mmWave-based 5G networks^10^.

Recently, the studies of a significant number of mmWave antennas have been published^11–26^. These antennas exhibit diverse structures based on their working principles, fabrication complexities, and cost considerations. Mainly the antennas are based on three techniques namely Substrate Integrated Waveguide (SIW), Cavity Back slot structures and simple planar antennas. In^11^ an mmWave dual-polarized beam-scanning SIW phased array antenna is discussed through Characteristic Mode Analysis. The antenna incorporates a differentially fed SIW cavity for horizontal polarization and a linearly tapered microstrip feed for vertical polarization, ensuring strong coupling while maintaining high isolation exceeding 31.5 dB. The 1 × 4 phased array achieves an 11.0% dual-polarization bandwidth from 25.8 to 28.8 GHz, with a return loss better than 10 dB. At 28 GHz, the array demonstrates a beam-scanning range of ± 44° for horizontal polarization and ± 41° for vertical polarization. Similarly, in^12^, a SIW array at 30 GHz is discussed. The array elements are arranged front-back, four on each side. For enhanced beam steering, a high-permittivity dielectric layer is placed below the radiating elements. In^13^, a compact dual-band dual-circularly polarized SIW cavity-backed antenna array is designed for mmWave applications. The proposed antenna utilizes a square SIW cavity with perturbation notches to generate right-hand circular polarization (RHCP) at 28 GHz and a dumbbell-shaped patch inside the aperture to achieve left-hand circular polarization (LHCP) at 38 GHz. In SIW, the bonding films and vias assembly make the antenna challenging to embed in RF circuits. A substrate Integrated cavity backed (SIC) antenna element is discussed in^14^ for mmWave resonance of 31.31–44.91 GHz. The reported structure utilizes the TM₂₁₁ mode within a SIC structure, enhanced by an H-shaped patch to improve impedance matching and radiation stability. The 4 × 4 array achieves an fractional bandwidth of 32.71% and a peak gain of 21.8 dBi at 40 GHz. The radiation patterns remain stable with low cross-polarization levels below − 30 dB. In^15^, a compact four-element mmWave planar array with modified chuck wagon bell shape is presented. The four element array has physical dimensions of 24 × 18.85mm^2^ while the bandwidth is 2.9 GHz with 28 GHz central frequency. A cavity backed dual-split-ring resonator (DSRR) planar structure is discussed in^16^ with dual band response at 28 and 38 GHz. The antenna incorporates three-layer PCB for better impedance matching and gain enhancement. The larger split ring resonator generates primary resonance with fractional bandwidth of 12.5%, while smaller generates 38 GHz resonance with 5% fractional bandwidth which performance is enhanced further by parasitic branches. The proposed structure is transformed into four element linear array network. A peak gain of 12.8 dBi is observed for 28 GHz and 14.9 dBi for 38 GHz. In^17^, a dual band mmWave antenna is presented at 28 and 38 GHz. The propose design combines dual microstrip loop (MDL) which contributes to primary resonance of 28 GHz and microstrip patch structure (MP) which generates second resonance at 38 GHz. A 2 × 1 antenna array of proposed design is developed, achieving wider bandwidths of 15.1% (25.7–29.9 GHz) and 7.4% (37.2–40 GHz) and higher gains of 13.1 dBi at 27 GHz and 12.3 dBi at 38 GHz. In^18,19^, a planar based array structures are discussed at 28 GHz resonance. The gain at both reported literature is observed to be < 10 dB.

This article introduces a compact dual-key shaped antenna that operates at dual-band frequencies mmwave frequencies for 5G services. The key highlights of the proposed work is as following.

- The proposed antenna design is developed using Characteristic Mode Theory (CMT) and operates at two mmwave 5G bands of 28 and 38 GHz.
- The proposed structure is further extended into a 1 × 4 linear array, improving overall performance.
- The array achieves peak gains of 10.5 dBi at 28 GHz and 11 dBi at 38 GHz and maintains a total efficiency above 75% across both operating bands.
- The complete array retains a compact size of 19.5 × 25 mm², suitable for space-limited 5G devices.
- Compared to existing mmWave designs, the proposed structure offers simpler geometry and improved performance, making it a strong candidate for 28/38-GHz 5G applications.

## Antenna design

### Single-element

**Fig. 1:**
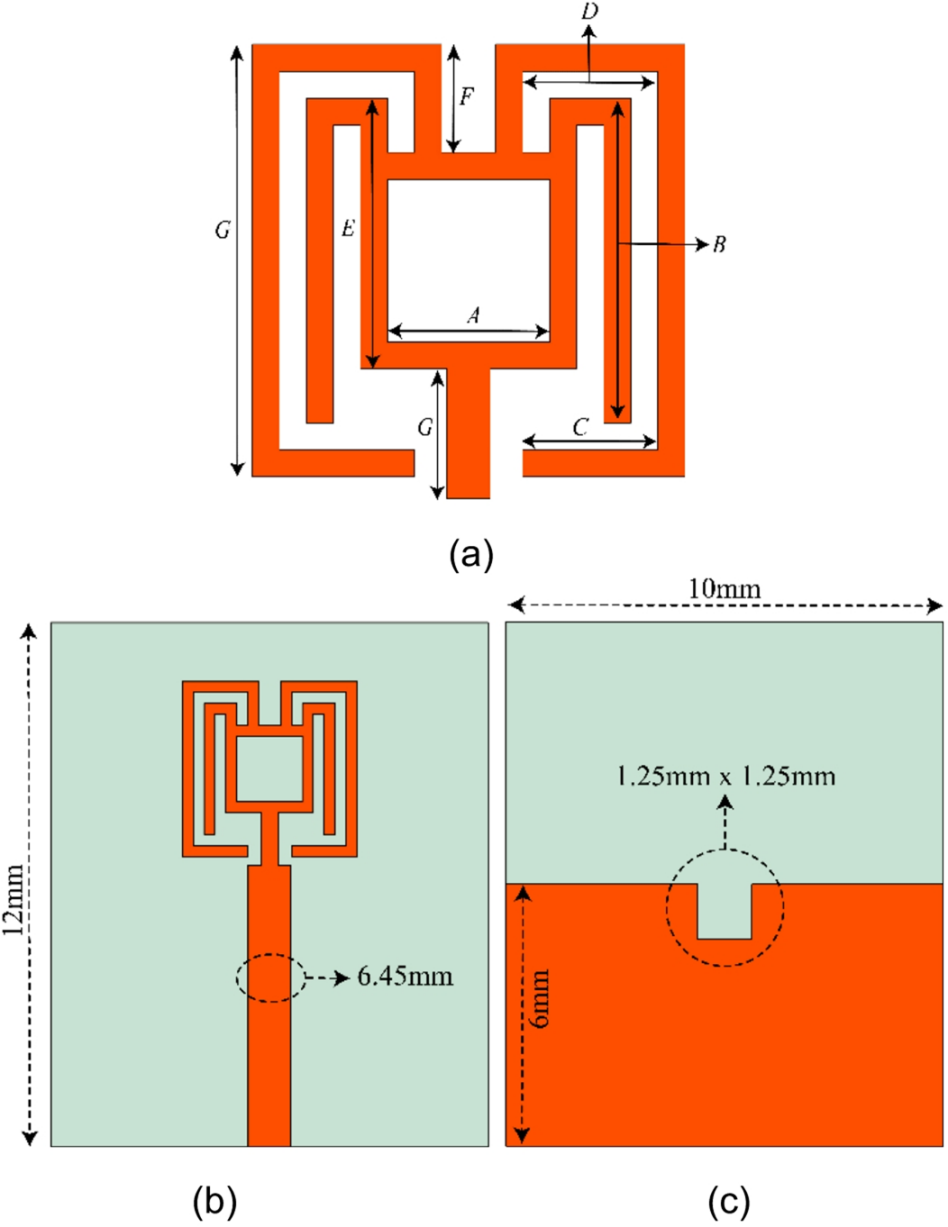
The proposed single element. (**a**) close view of meandered section patch (**b**): Complete Front view. (**c**): Back view.

The proposed dual key shape antenna is designed on ultra-thin 0.254 mm RO5880 board with permittivity value of 2.2. Figure 1 shows the proposed antenna. The physical size is noted as 12 mm × 10 mm, featuring a dual-key shaped radiator with a central square slot and a meandered structure for resonance tuning. A partial 6 mm ground plane, incorporates a small rectangular slotted notch of 1.25 mm × 1.25 mm to enhance impedance matching and bandwidth. Table 1 shows complete physical dimensions of proposed structure.

**Table 1:** Physical parameters of proposed antenna in mm.

Parameter	A	B	C	D	E	F	G
Value	1.5	3	1.25	1.25	3.5	1	4

**Fig. 2:**
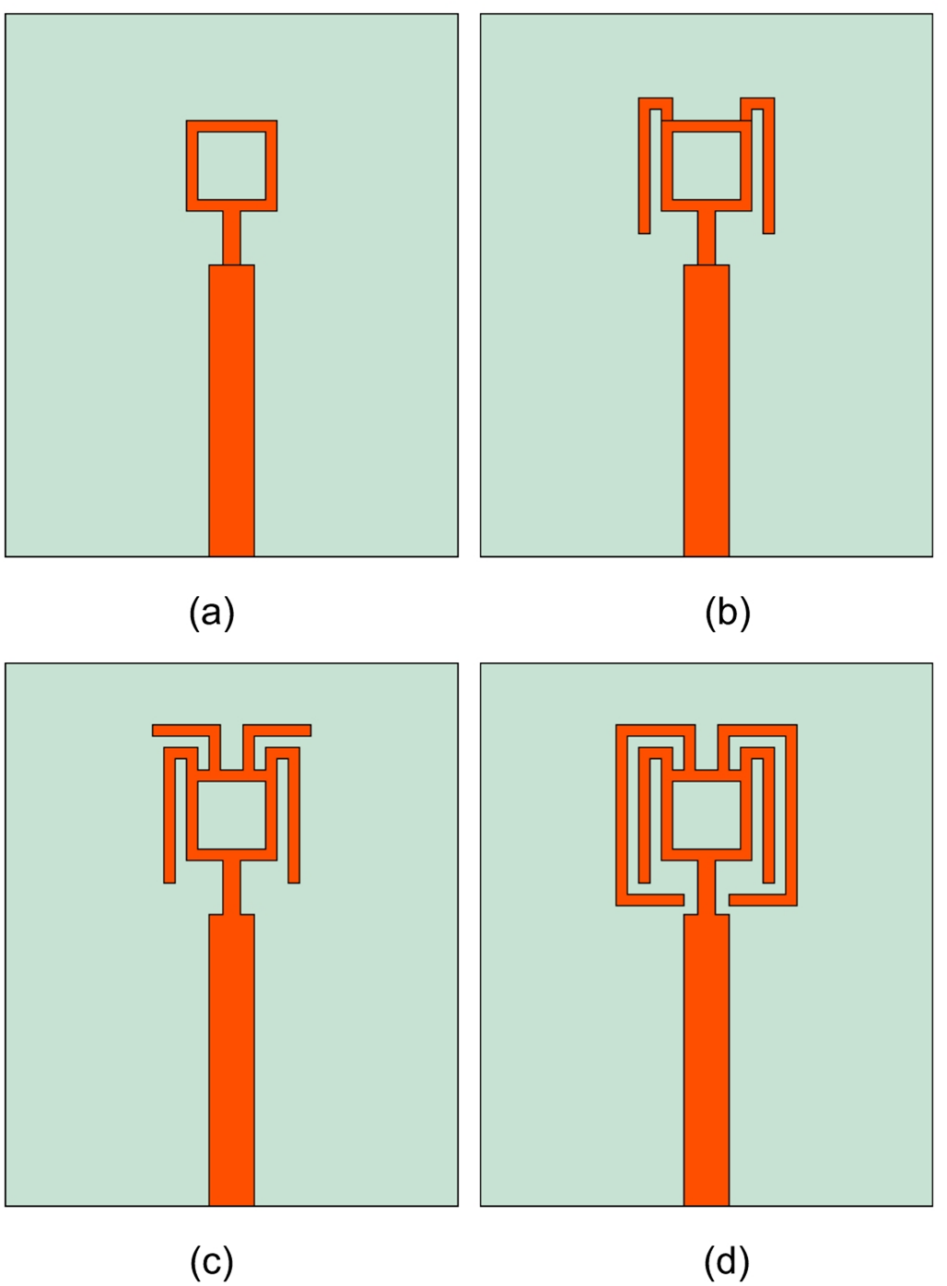
Evolution Stage. (**a**) 1 (**b**) 2 (**c**) 3 (**d**) 4.

The single-element antenna is evolved in four stages to achieve dual-band resonance at 28 and 38 GHz. Figure 2 cover the design evolution steps. The approach taken for evolution stages is characteristic mode theory (CMT). The CMT is used to study the radiation characteristics and the conduction properties of complex structures where the defined numerical methods are unfeasible to apply. The CMT does not apply the port excitation; instead, it incident the tangential electric field $\:{(E}^{i})$ on to the conducting surface, due to which the surface currents $\:{(J}_{n})$ are generated. These surface currents are also termed as characteristic currents/modes obtained as the eigenfunction $\:\left({\lambda\:}_{n}\right)$, and are related as Eq. (1)^1^,

1 $$\:X\left({J}_{n}\right)=\:{\lambda\:}_{n}R\left({J}_{n}\right)$$

Where $\:R$ and $\:X$ are the real and imaginary parts of the impedance operator $\:Z$, which is $\:Z=R+jX$. The surface currents $\:{(J}_{n})$ give rise to electric field $\:\left({E}^{S}\right)$ on the conducting surface called the characteristic field, which are derived from impedance operator $\:Z$ as,

2 $$\:{E}^{S}=Z\left({J}_{n}\right)=R\left({J}_{n}\right)+jX\left({J}_{n}\right)$$

Substituting for $\:X$ from Equaiton (1),

3 $$\:{E}^{S}=R\left({J}_{n}\right)\left(1+j{\lambda\:}_{n}\right)$$

The total current $\:\left(J\right)$ is the summation of mode weighted coefficient $\:{\alpha\:}_{n}$, with characteristic mode $\:{J}_{n}$, that is Eq. (4)^2^,

4 $$\:J = \:\sum {{\:_n}} \alpha {\:_n}{J_n} = \:\frac{{\left\langle {{E^i},\:{J_n}} \right\rangle }}{{\left( {1 + j\lambda {\:_n}} \right)}}\:\:{J_{n\:}}$$

The model weighted coefficient $\:{\alpha\:}_{n}$ projects the contribution of each mode. It is directly related to the amount of field excitation $\:{E}^{i}$ to the specific mode and it is maximum when $\:{\lambda\:}_{n}=0$. The $\:{\lambda\:}_{n}$ is different for different structures. Therefore, the CST simulation tool is used to excite the structure through an incident wave, and the structure determines the mode’s $\:{\lambda\:}_{n}$, and thereafter $\:{\alpha\:}_{n}$ is computed.

The modal significance (MS) is the derivative of total current $\:J$, which reflects the normalized amplitude of the modes, that is $\:\left|\frac{1}{(1+j{\lambda\:}_{n})}\right|$. When the eigenvalue $\:{\lambda\:}_{n}=0$, the mode amplitude is 1, indicating the radiating mode. More physical insight is obtained when the phase difference between the modes is studied through characteristic angles, which is defined as,

5 $$\:{CA}_{n}=180^\circ\:-{tan}^{-1}\left({\lambda\:}_{n}\right)$$

**Fig. 3:**
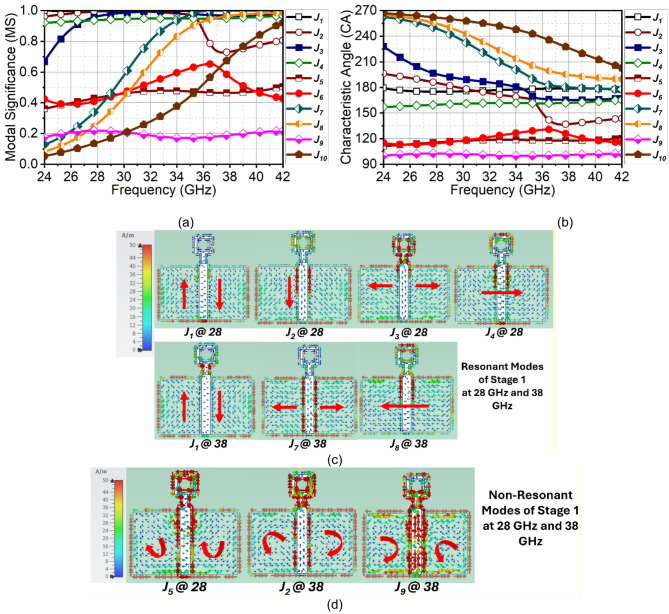
CMT results of evolution stage-1. (**a**) MS, (**b**) $\:{CA}_{n}$, (**c**) Surface current of resonant modes and (**d**) non-resonant modes.

For positive $\:{\lambda\:}_{n}$, the $\:{CA}_{n}$ ranges from < 180° and > 90°, representing the mode is inductive. On the other hand, for negative $\:{\lambda\:}_{n}$, the $\:{CA}_{n}$ ranges from > 180° and < 270°, indicating the capacitive mode. For both cases, the antenna structure stores the energy in the near-field region, which results in lower radiation efficiency $\:{\eta\:}_{rad}$. By changing the antenna structure, the characteristic modes changes, which are also frequency-dependent, may aid in attaining the zero eigenvalue $\:{\lambda\:}_{n}$, by which the $\:{CA}_{n}$ converges to 180°. Thus, the mode is turned into a radiating mode, and the maximum energy is radiated. For our structure analysis, we considered ten modes for each of the evolution stages to comprehend the significant and contributing modes. The proposed structure has multiple open-ended bend conducting lines connected to a rectangular ring. From the basic theory of antennas, a bend in the transmission line with matched impedance and an approximate bend length of half-wavelength will generate radiation, leading to a simple dipole structure. However, the proposed structure involves multiple bends and multiple arms. Thus, the analytical value from the conventional theoretical method is not appropriate; as a result, the CMT is used, which provides insight into surface currents for any geometrical structure.

Generally, the first five modes for most structures determine the resonant modes; however, in our case, some higher-order modes contribute to the resonance. As a result, ten modes are considered for the analysis. The first stage is a rectangular ring with partial ground; in such case of partial ground, the conducting surface on top and bottom are both responsible for radiation. Consequently, a multilayer solver is used for the analysis. The MS results in Fig. 3(a) indicate, that the Modes $\:{J}_{1}$ to $\:{J}_{4}$ are significant at 28 and 38 GHz. Additionally, the Modes $\:{J}_{7}$ and $\:{J}_{8}$ are also significant at 38 GHz. Theoretically, all these modes must converge at 180° or have equi-phase differences to achieve radiation and resonances. However, observing $\:{CA}_{n}$ in Fig. 3(b), though the above modes converge and have an equi-phase difference, there are specific modes that have non-equi-phase differences, and as a result, large energy is held in the near-field. The Modes $\:{J}_{1}$ to $\:{J}_{4}$ are converging at 28 GHz, at the same time Modes $\:{J}_{5}$ to $\:{J}_{10}$ are inductive and capacitive with unequal phase difference, causing antenna to be non-radiative. A similar effect is also observed at 38 GHz with converging Modes of $\:{J}_{1}$ and $\:{J}_{7}$, and partially $\:{J}_{8}$. The remaining modes are non-convergent and non-radiative. The resonant modes form longitudinal and transversal surface currents on the structure. On the other hand, the non-resonant modes generate loop currents, resulting in energy storage in the near-field. The converging Modes $\:{J}_{1}$ to $\:{J}_{4}$ depicts longitudinal and transversal currents at 28 GHz. Similar behavior of $\:{J}_{1}$, $\:{J}_{7}$, and $\:{J}_{8}$ is depicted at 38 GHz in Fig. 3(c). However, the remaining modes are non-convergent modes with loop current; due to brevity, only a few of these modes are illustrated in Fig. 3(d). As explained before, due to unequal phase difference of non-convergent modes, the stage 1 antenna is non-radiative.

**Fig. 4:**
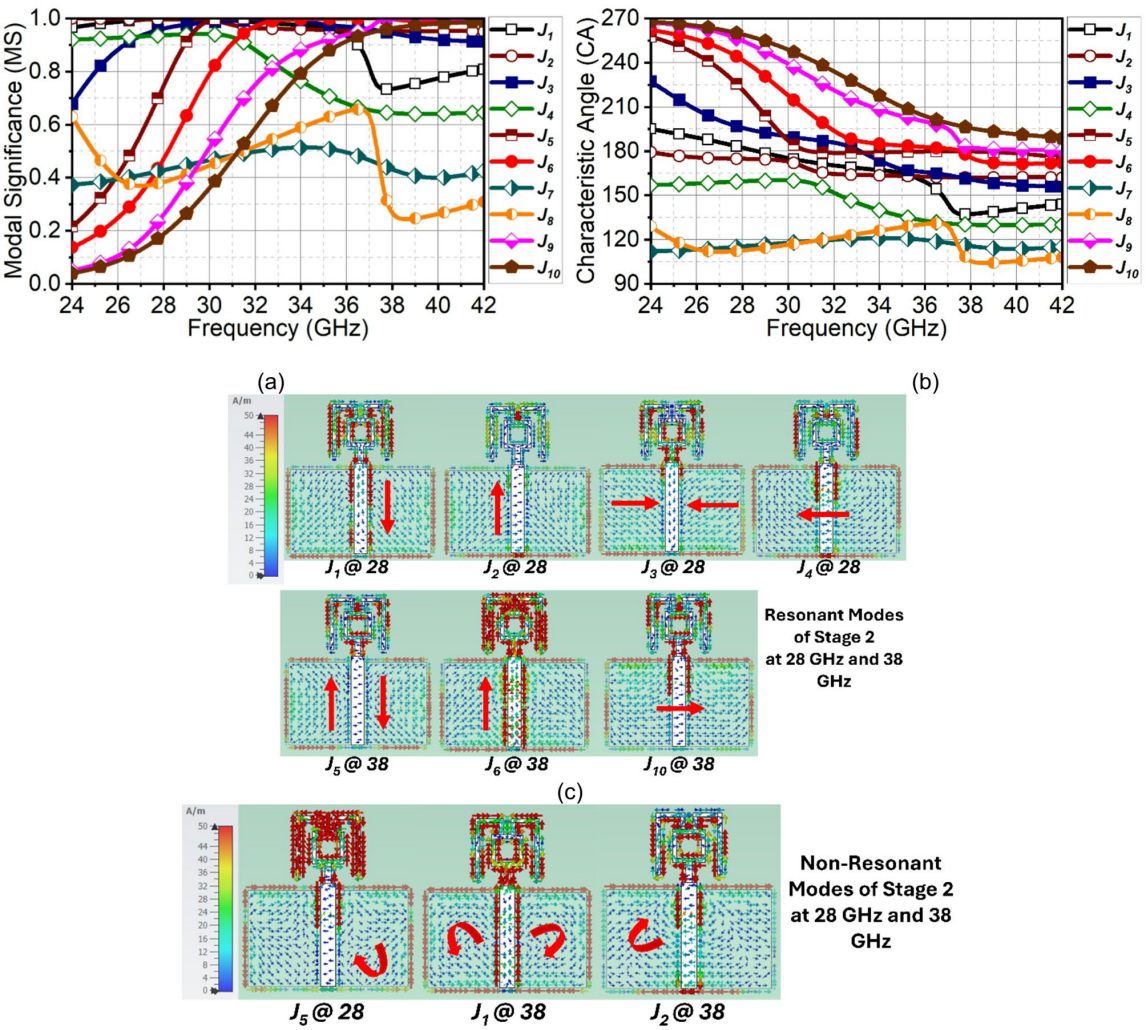
CMT results of evolution stage-2. (**a**) MS, (**b**) $\:{CA}_{n}$ (**c**) Surface current of resonant modes and (**d**) non-resonant modes.

In the next evolution stage, two bended conducting strip lines are added to the ring, which is approximately $$\:\raisebox{1ex}{${\lambda\:}_{1}$}\!\left/\:\!\raisebox{-1ex}{$2$}\right.$$ long (where $$\:{\lambda\:}_{1}$$ is wavelength at 28 GHz). The modification to the structure has changed the mode of operation of modes. Now the Modes $$\:{J}_{1}$$ to $$\:{J}_{4}$$ attain normalized amplitude close to 1 at 28 GHz, however, the Modes $$\:{J}_{1}$$ and $$\:{J}_{4}$$ are suppressed at 38 GHz, as illustrated in Fig. 4(a). Instead, the Modes $$\:{J}_{5}$$, $$\:{J}_{6}$$, and $$\:{J}_{10}$$ attains significance at 38 GHz. The modification to the structure has resulted in reduced phase difference between the modes, as depicted in Fig. 4(b), however, it is not a significant change. The same can be validated through the current distribution in Fig. 4(c) and 4(d).

**Fig. 5:**
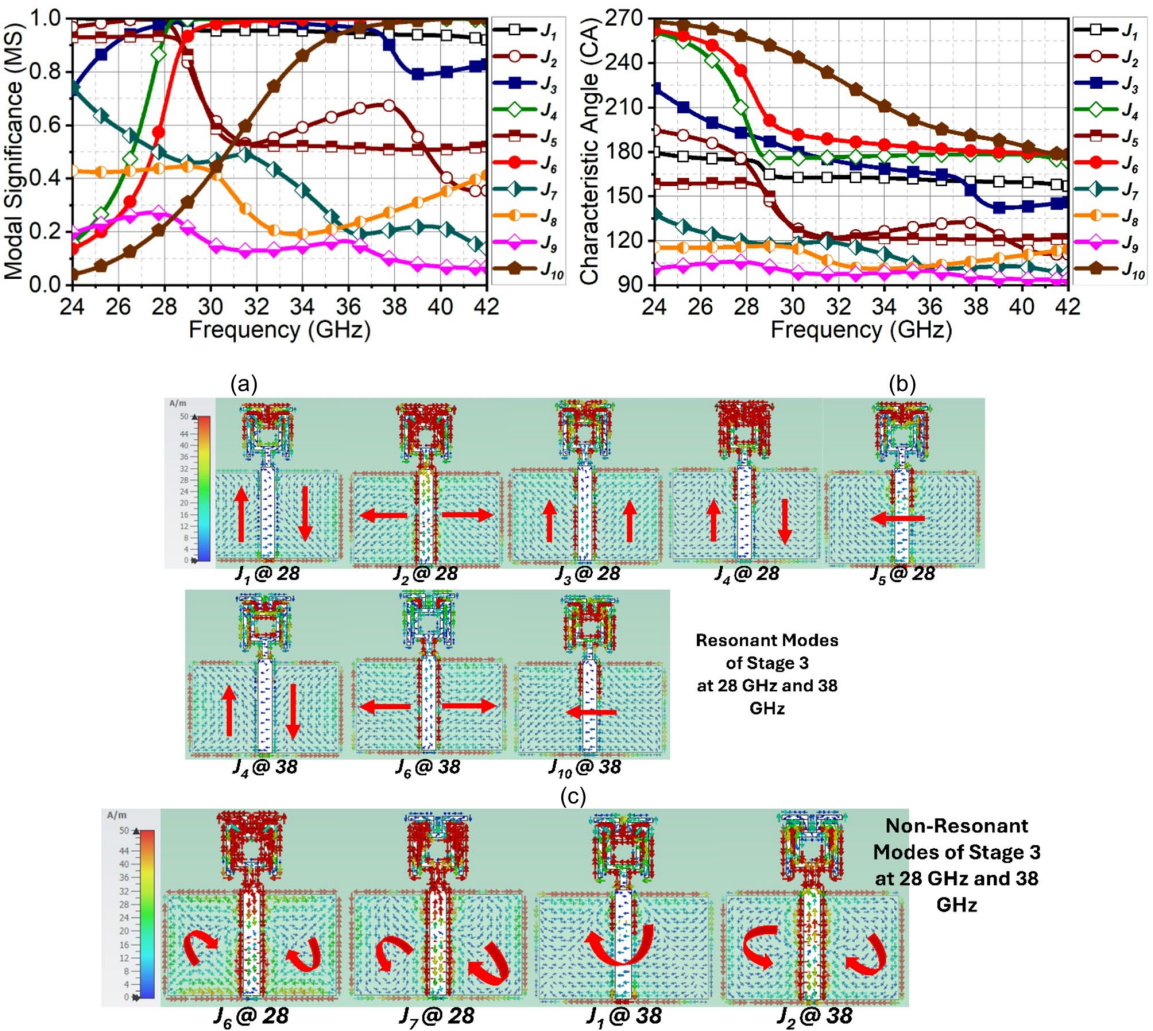
CMT results of evolution stage-3. (**a**) MS, (**b**) $\:{CA}_{n}$ (**c**) Surface current of resonant modes and (**d**) non-resonant modes.

In stage-3, the spacing between strip lines and the ring is reduced, and another set of strip lines of $\:0.17{\lambda\:}_{1}$ long are added to the ring to reduce the capacitance effect by inductive loading. This has significantly changed the mode characteristics at 28 GHz than the 38 GHz resonance. For example, here, the Mode $\:{J}_{2}$ which was significant at both the resonance in the previous stage is not suppressed at the second resonance, whereas Mode $\:{J}_{1}$ is significant in both bands. At first resonance, Modes $\:{J}_{1}$ to $\:{J}_{5}$ attain the highest normalized amplitude, as in Fig. 5(a). On the other resonance, $\:{J}_{1}$, $\:{J}_{4}$, $\:{J}_{6}$, and $\:{J}_{10}$ are significant. Observing the phase difference in Fig. 5(b), indicates the Modes $\:{J}_{1}$, $\:{J}_{2}$ and $\:{J}_{4}$ (where $\:{J}_{4}$ is quasi-mode as depicted by surface current) converge to 180°, whereas Mode $\:{J}_{3}$ and $\:{J}_{5}$ are complex conjugate of each other, attaining equi-phase difference at first resonance. In this structure, Modes $\:{J}_{7}$ to $\:{J}_{9}$ are insignificant and can be neglected. Discussion on second resonance, the Modes $\:{J}_{4}$, $\:{J}_{6}$, and $\:{J}_{10}$ converge, but due to inductive phase of 15°, 40°, and 45° of Modes $\:{J}_{1}$, $\:{J}_{3}$, and $\:{J}_{2}$, the second resonance cannot be achieved. Figure 5(c) and 5(d) illustrates the resonant and nont-resonant modes at 28 and 38 GHz.

**Fig. 6:**
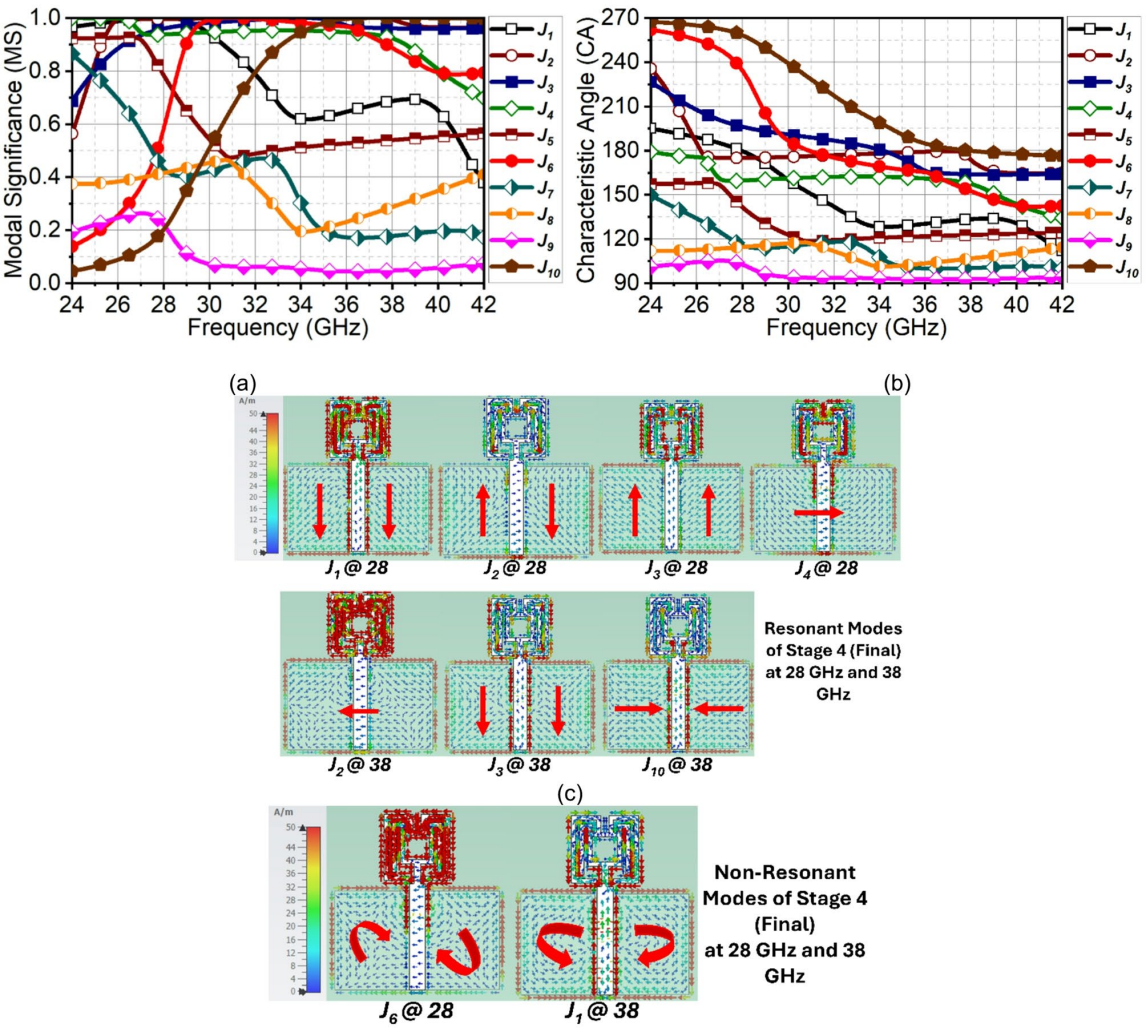
CMT results of evolution stage-4. (**a**) MS, (**b**) $\:{CA}_{n}$, (**c**) Surface current of resonant modes and (**d**) non-resonant modes.

In the final stage, the pair of strip lines, which were $\:0.17{\lambda\:}_{1}$ are extended to $\:0.67{\lambda\:}_{1}$ long, this is to improve the capacitive loading counteracting the inductive loading of the previous stage. This modification has suppressed the undesired Modes $\:{J}_{5}$, $\:{J}_{7}$, $\:{J}_{8}$, and $\:{J}_{9}$. The modes in the final stage contributing to resonance are the summation of $\:{J}_{1}+{J}_{2}+{J}_{3}{+\:J}_{4}$ at the first resonance and $\:{J}_{2}+{J}_{3}+{J}_{10}$ at the second resonance, as observed from Fig. 6(a). Observing the phase difference, Modes $\:{J}_{1}$ and $\:{J}_{2}$ converge at 180°, similar to the earlier stage with $\:{J}_{3}$ and $\:{J}_{4}$ as complex conjugate modes at first resonance. At the second resonance, Modes $\:{J}_{2}$ and $\:{J}_{10}$ converge at 180° and Modes $\:{J}_{3}$, $\:{J}_{4}$, and $\:{J}_{6}$ converge with a 15° phase difference, which is negligible, as depicted in Fig. 6(b). To support this analysis, the surface current in Fig. 6(c) indicates a longitudinal and transversal current of $\:{J}_{1}$, $\:{J}_{2}$, $\:{J}_{3}$, and $\:{J}_{4}$ at 28 GHz, and the combination of these currents leads to antenna resonance. Similarly, at 38 GHz, $\:{J}_{2}$, $\:{J}_{3}$, and $\:{J}_{10}$ constitute transversal and longitudinal currents causing a second resonance. On the other hand, the non-contributing modes constitute loop current as shown in Fig. 6(d).

**Fig. 7:**
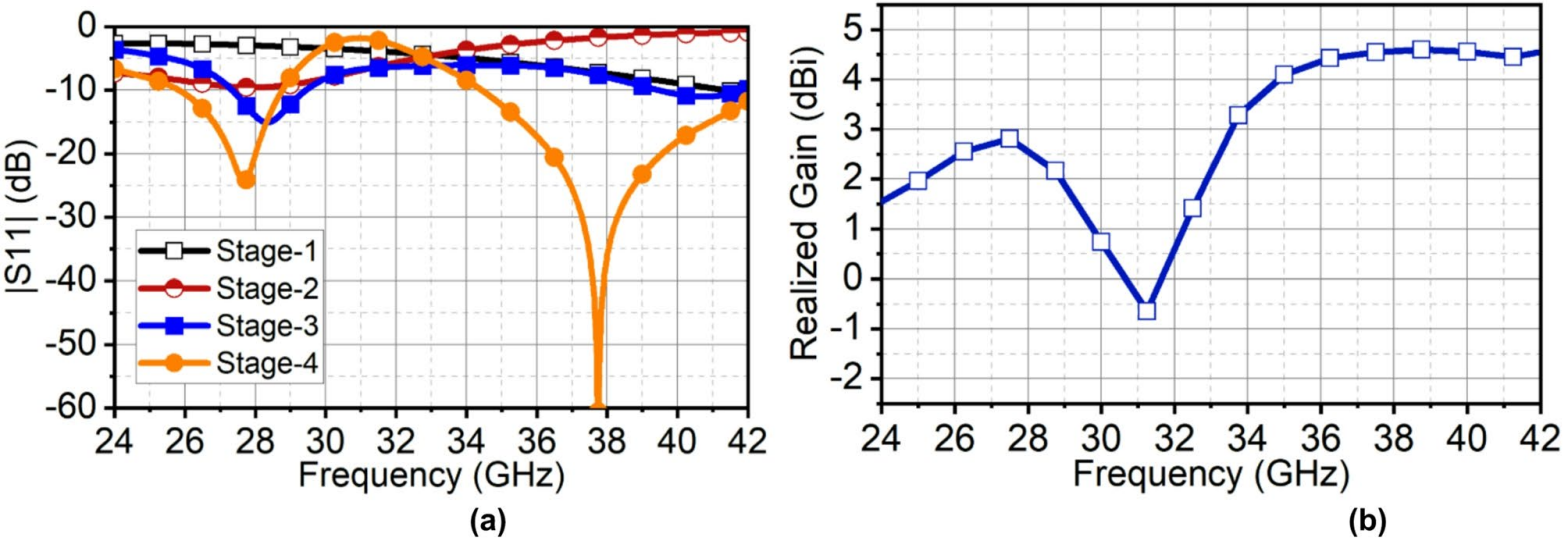
(**a**) |S11| of design evolution (**b**) Realized gain of the proposed single-element antenna.

To comprehend the analysis, all the evolution stages are excited by the port, and its reflection coefficient response is shown in Fig. 7. The results in Fig. 7 indicate that stage 3 has achieved the resonances at first and second band, however, with low impedance matching. Further, extending the length of strip lines has improved the capacitive loading and suppressed the inductive Modes, leading to optimum impedance matching at both bands. The gain of this antenna is low; thus, to improve the gain and directivity, the antenna is extended to a linear array of $\:1\times\:4$ elements, which will be discussed in the next section. It is also worth noting that the antenna has filter characteristics out of the band.

### Array configuration

**Fig. 8:**
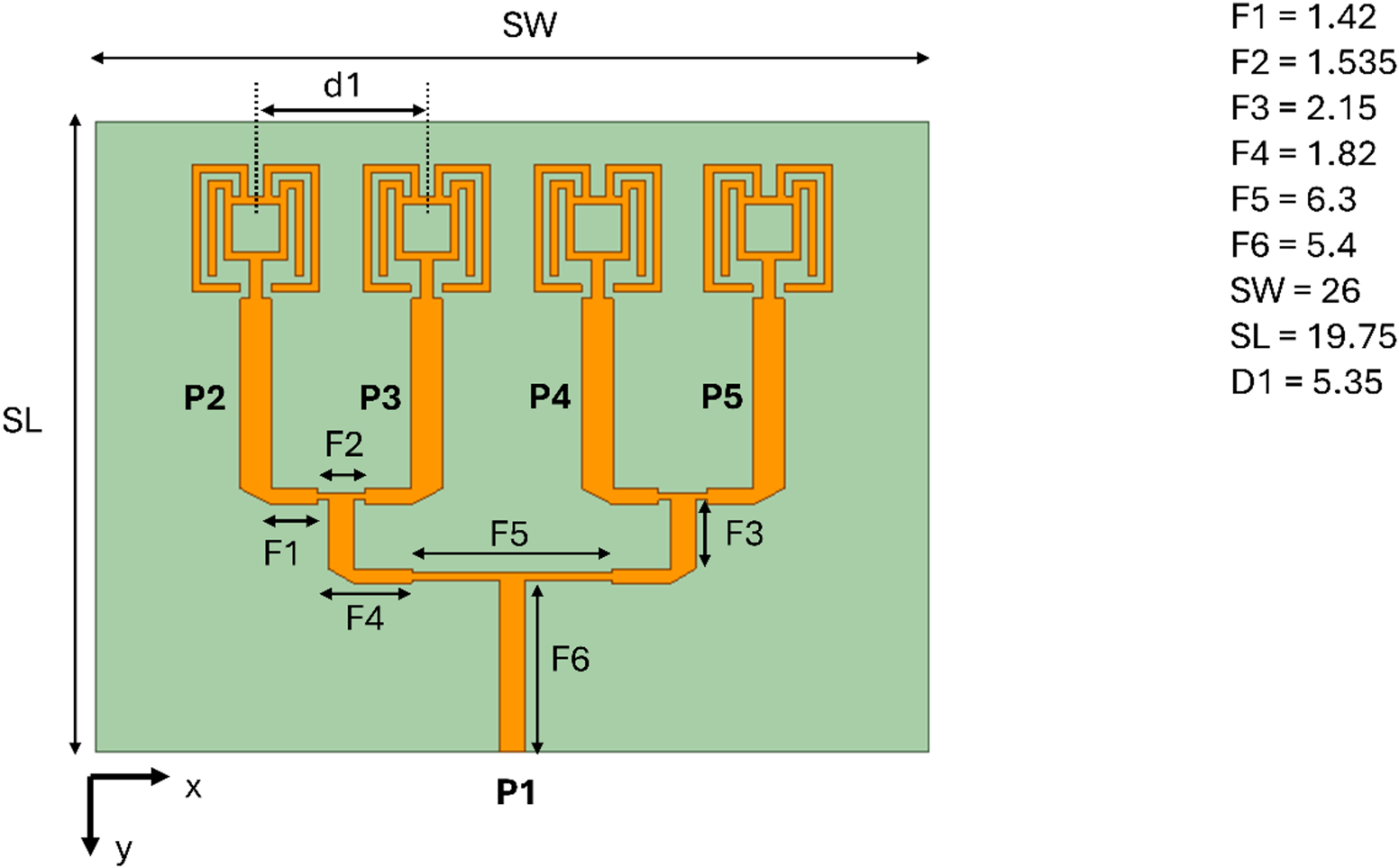
Proposed array antenna structure. The structure’s dimension in mm is as follows: F1 = 1.42, F2 = 1.535, F3 = 2.15, F4 = 1.8, F5 = 6.3, F6 = 5.4, SW = 26, SL = 19.75, and d1 = 5.35.

**Fig. 9:**
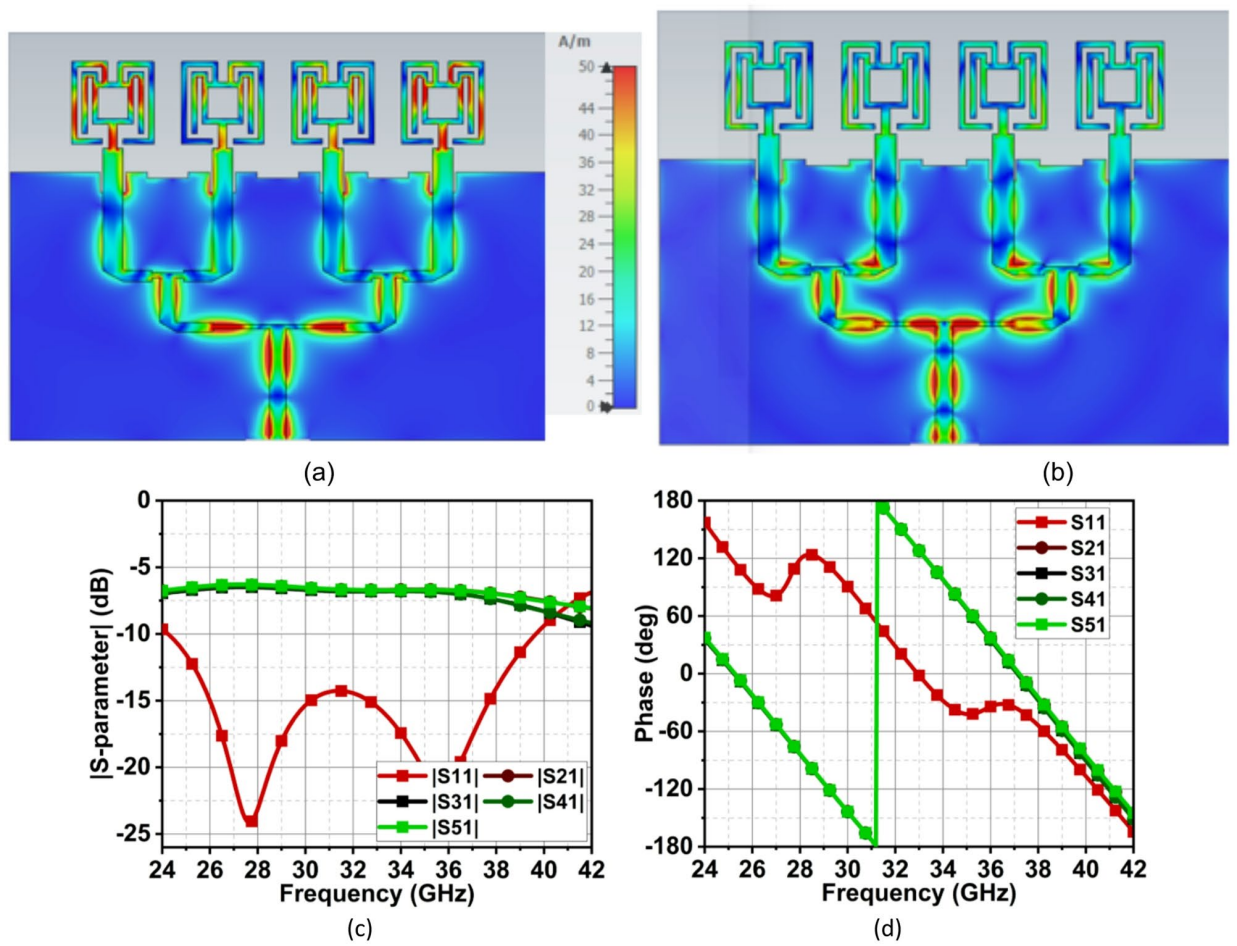
Surface current representation of proposed array antenna structure at (**a**) 28 GHz and (**b**) 38 GHz. (**c**) Feed network power distribution and (d) phase across each radiating element.

As discussed earlier, the single-element antenna is expanded to a $\:1\times\:4$ linear array along the x-axis. The inter-element spacing (*d1*) between the elements is kept at $\:0.5{\lambda\:}_{1}$, such that a constructive narrow beam is formed in the xz-plane. The simple T-junction power divider makes the proposed structure easily scalable to $\:1\times\:8$ or $\:1\times\:16$ configuration. This will increase the gain, directivity, and bandwidth as well. However, as the number of T-junction power dividers increases, the feedline losses also increase, and the antenna performance may degrade at some point. So it is a trade-off between the number of radiating elements and performance. Therefore, the design in this article is restricted to $\:1\times\:4$ array configuration. The overall dimension of array antenna is $\:2.43{\lambda\:}_{1}\times\:1.84{\lambda\:}_{1}$, which is compact and suitable for many mmWave applications. To form the broadside beam at $\:\theta\:=0$°, all the radiating elements must receive equal power and phase, thus, a two-level corporate feed is used. To match the impedance of the radiating element feed, which is of 43 Ω, with the 100 Ω feed line of $\:F2,$ the $\:F1$impedance is maintained at 65 Ω with a strip length of $\:0.135{\lambda\:}_{1}$long. Here, impedance of $\:F1=\:\sqrt{F0\times\:F2}$, where $\:F0$ is the impedance of the radiating element. Further to match the impedance of $\:F2$, a 50 Ω feed line $\:F3$ is used, which splits the power equally to $\:P2$ and $\:P3$. The same structure is replicated by separating with a half-wavelength, constituting two $\:1\times\:2$ array antennas. These two array antennas are further connected with another T-junction power divider with $\:F4$, $\:F5$, and $\:F6$ having an impedance of 70.7 Ω, 100 Ω, and 50 Ω, as shown in Fig. 8. To demonstrate the effective design of the feed network, the surface current distribution along with power and phase distribution to elements is shown in Fig. 9.

**Fig. 10:**
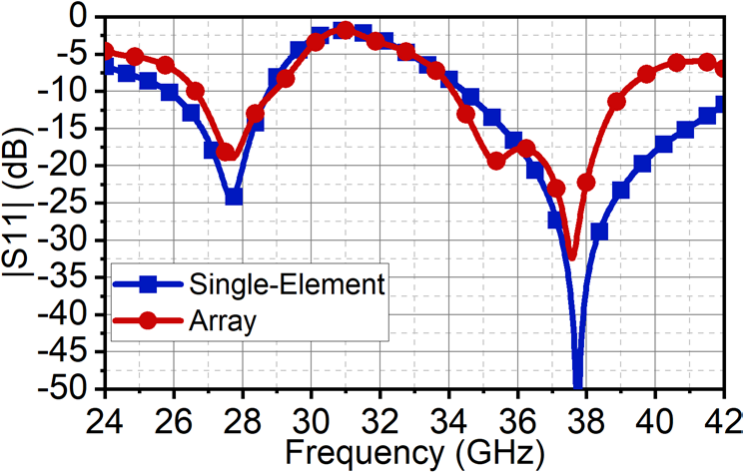
Comparison of reflection coefficient |S11| of array antenna with single-element antenna.

**Fig. 11:**
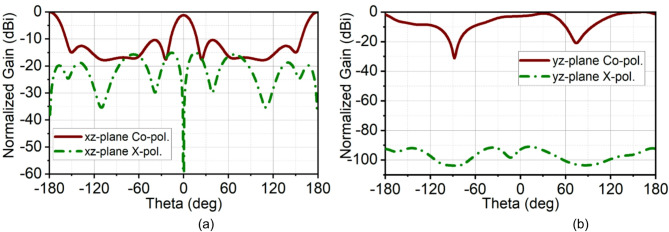
Simulated radiation pattern of array antenna at 28 GHz. (**a**) xz-plane, and (**b**) yz-plane.

The reflection coefficient |S11| at 10 dB is compared between the single-element and array antenna in Fig. 10. The array results show the design of the feed network, with the slight modification to the ground plane, has achieved exact resonance as of the single-element antenna; however, with the slight compromise in the bandwidth at the second band. It can be observed that the bandwidth of the array antenna at the first band is from 26.6 to 28.9 GHz, whereas for single-element, it is from 25.85 to 28.75 GHz, as shown in Fig. 10.

**Fig. 12:**
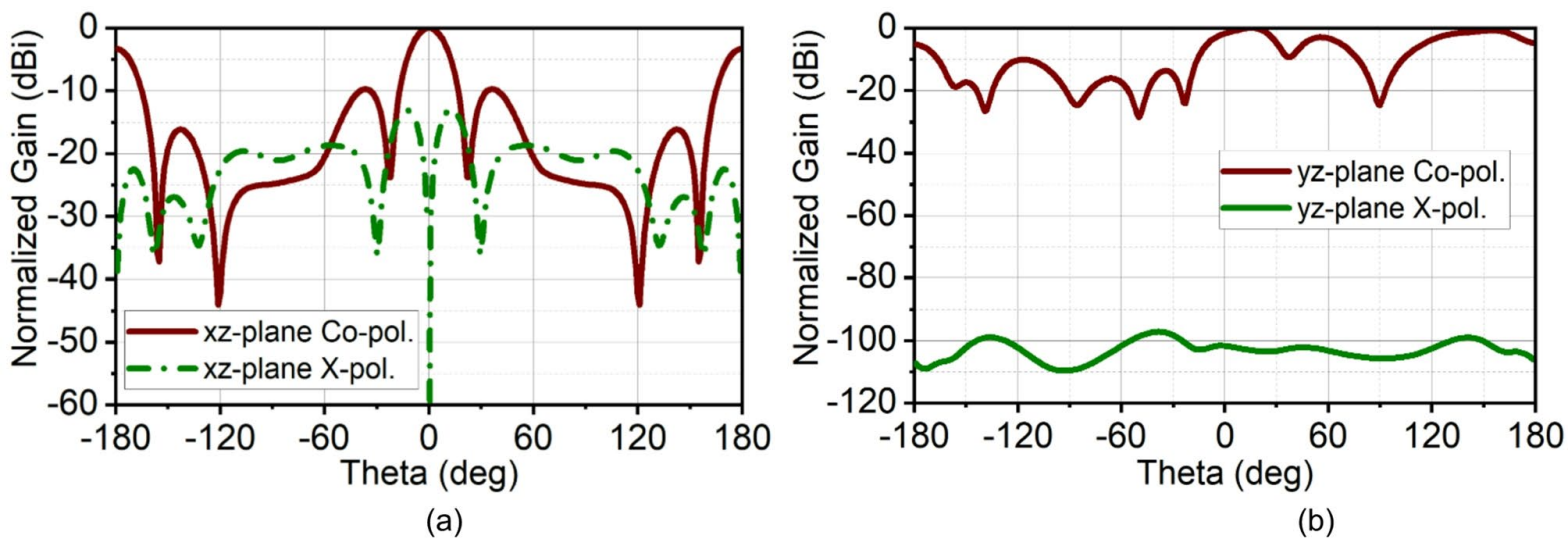
Simulated radiation pattern of array antenna at 38 GHz. (**a**) xz-plane, and (**b**) yz-plane.

**Fig. 13:**
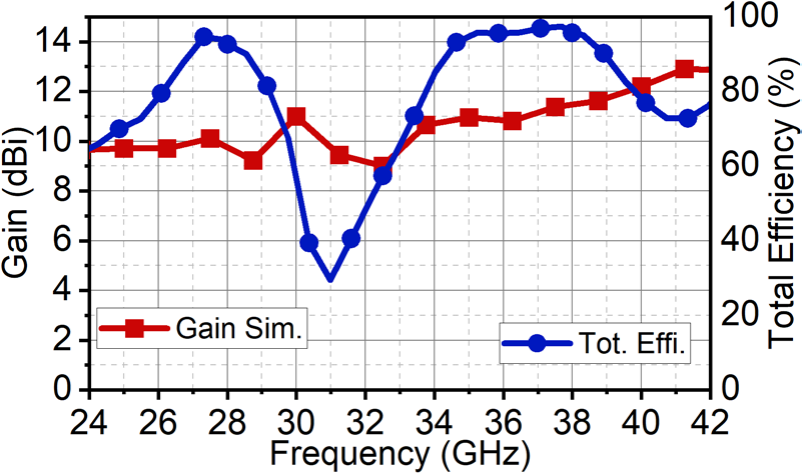
Simulated gain and total efficiency of the proposed array antenna.

This difference in bandwidth at the mmWave spectrum is negligible. It can be seen that in the second band, the array bandwidth range is 34.1–39.1 GHz, while the single-element bandwidth range is 34.45–42.5 GHz. This is because, in the pursuit of achieving good impedance matching at the second band, the ground structures are tuned, which results in reduced bandwidth, with the achieved fractional bandwidth of 7.26%. Nonetheless, the proposed structure offers good bandwidth with good directivity. As the radiating elements are arranged along the x-axis, a narrow beam pattern can be observed in the xz-plane (H-plane), and the orthogonal plane has a broad beam in the yz-plane (E-plane). The array antenna has resulted in a half-power beamwidth (HPBW) of 17° in the xz-plane with a minimum side-lobe level (SLL) of −10 dB and X-polarization of −15 dB at 28 GHz. In the orthogonal yz-plane, the beam is wide with HPBW of 54° with excellent X-polarization of −90 dB, as illustrated in Fig. 11. Similarly, at 38 GHz, the radiation has a narrow beam of 20° in xz-plane with SLL of −10 dB and X-polarization of −13 dB. In the yz-plane, the HPBW is 32.5° with a high X-polarization of −97 dB, as shown in Fig. 12. In the first band, the antenna has achieved a maximum gain of 10 dBi, and in the second, it is 11.5 dBi with 90% average total efficiency in both bands in Fig. 13. The antenna also demonstrates good filter response out-of-the-band, as observed from the total efficiency response.

## Results and discussison

**Fig. 14:**
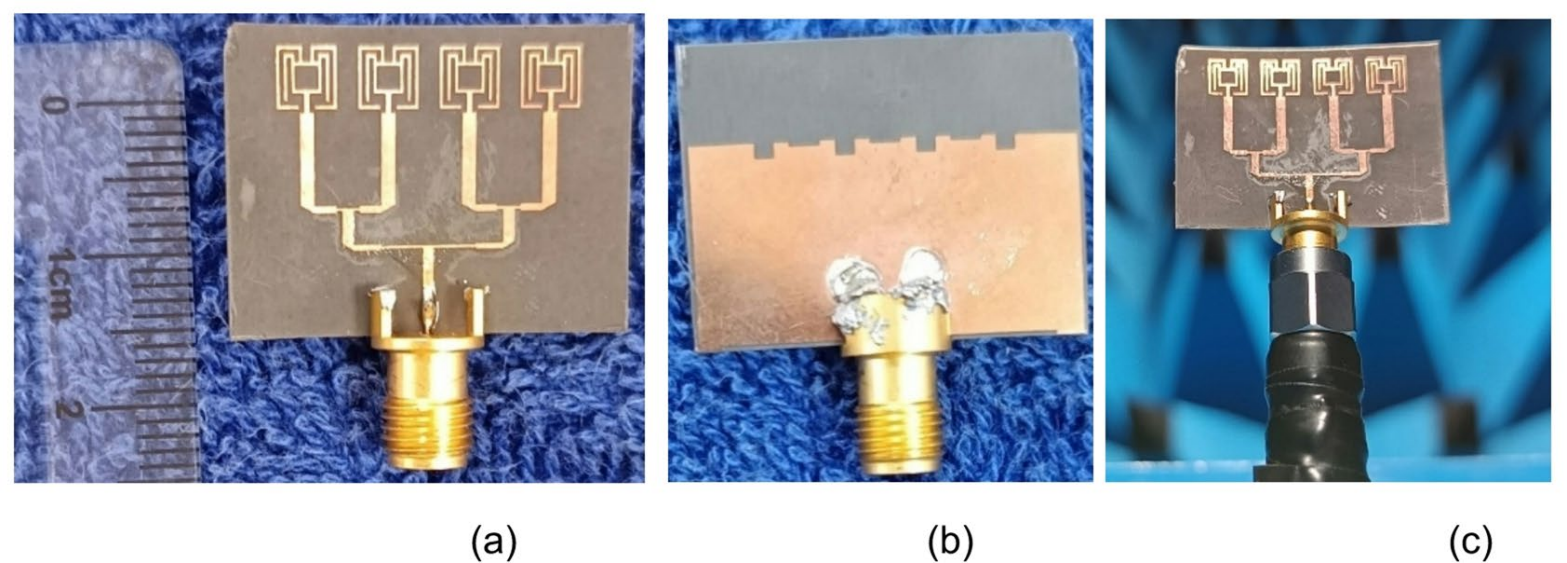
Prototype fabricated antenna is shown with a top view in (**a**) and (**b**) bottom (**c**).Radiation pattern measurement setup in an anechoic chamber.

To improve the gain and directivity, the single-element antenna is expanded to a linear array of four elements along the x-axis. With the proper element spacing the array has achieved better directivity and gain in the broadside direction. The radiating elements are fed with equal power and phase for which the corporate feed technique is used. The simulated results of the array antenna are validated by measuring the prototype antenna. The prototype fabricated antenna is shown in Fig. 14. The precision of the fabrication machine is 4 mils, which is equal to 0.1 mm. Consequently, the tracks and gap in the proposed antenna structure is > 0.1 mm to avoid the tolerance error. Also, a variation in 0.1 mm thickness or spacing will significantly affect the antenna performance at millimeter wave, resulting in a resonance and gain performance shift. Therefore, extreme care is taken during the design and fabrication process.

The signal from the vector network analyzer (VNA) (S820E) is coupled to the antenna through a 2.92 mm SMA connector which has an operating range of up to 40 GHz. The radiation pattern measurement setup in an anechoic chamber is shown in Fig. 14(c).

**Fig. 15:**
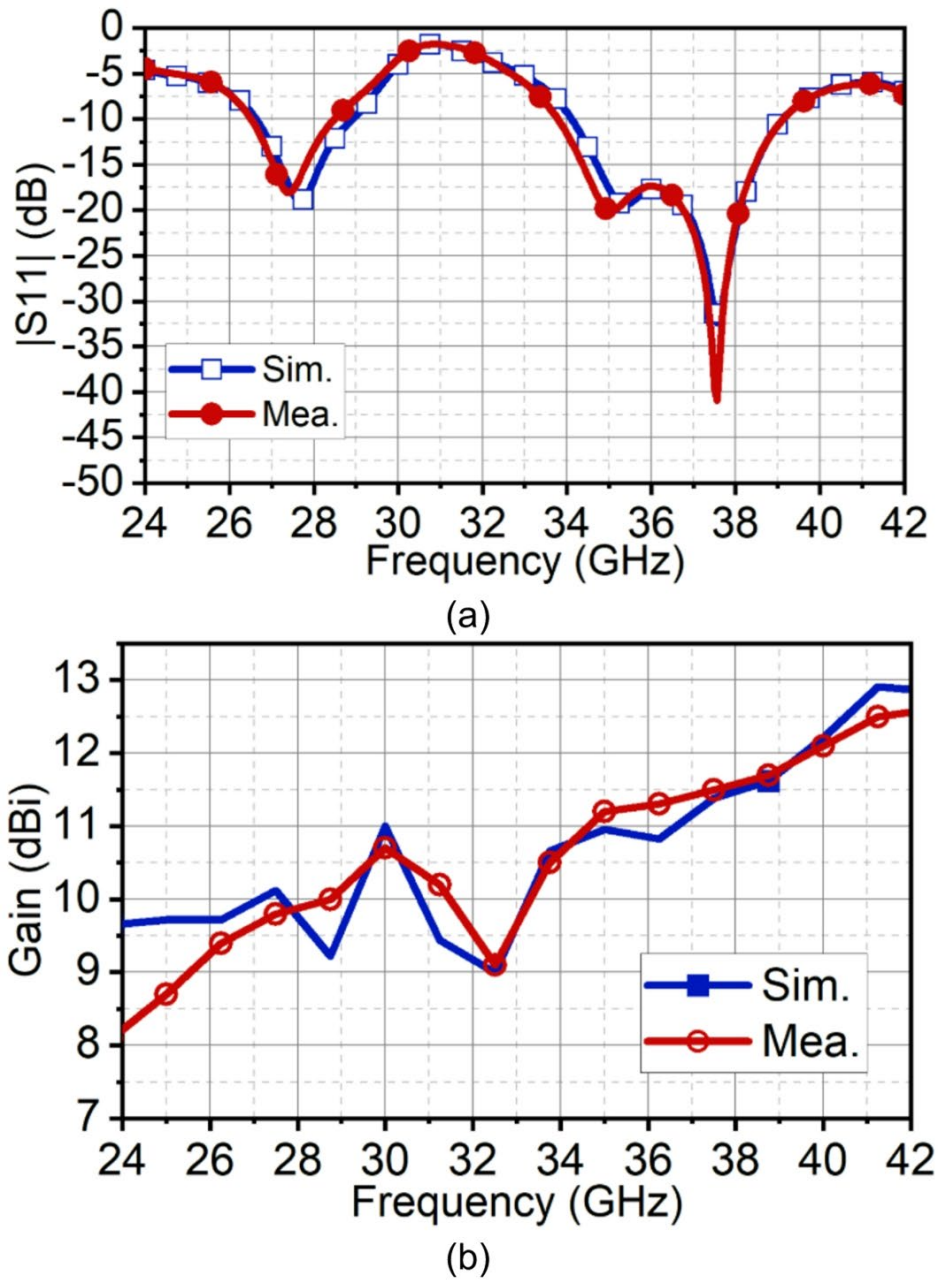
Simulated and measured (**a**) reflection co-efficient |S11| and (**b**) gain the proposed array antenna.

**Fig. 16:**
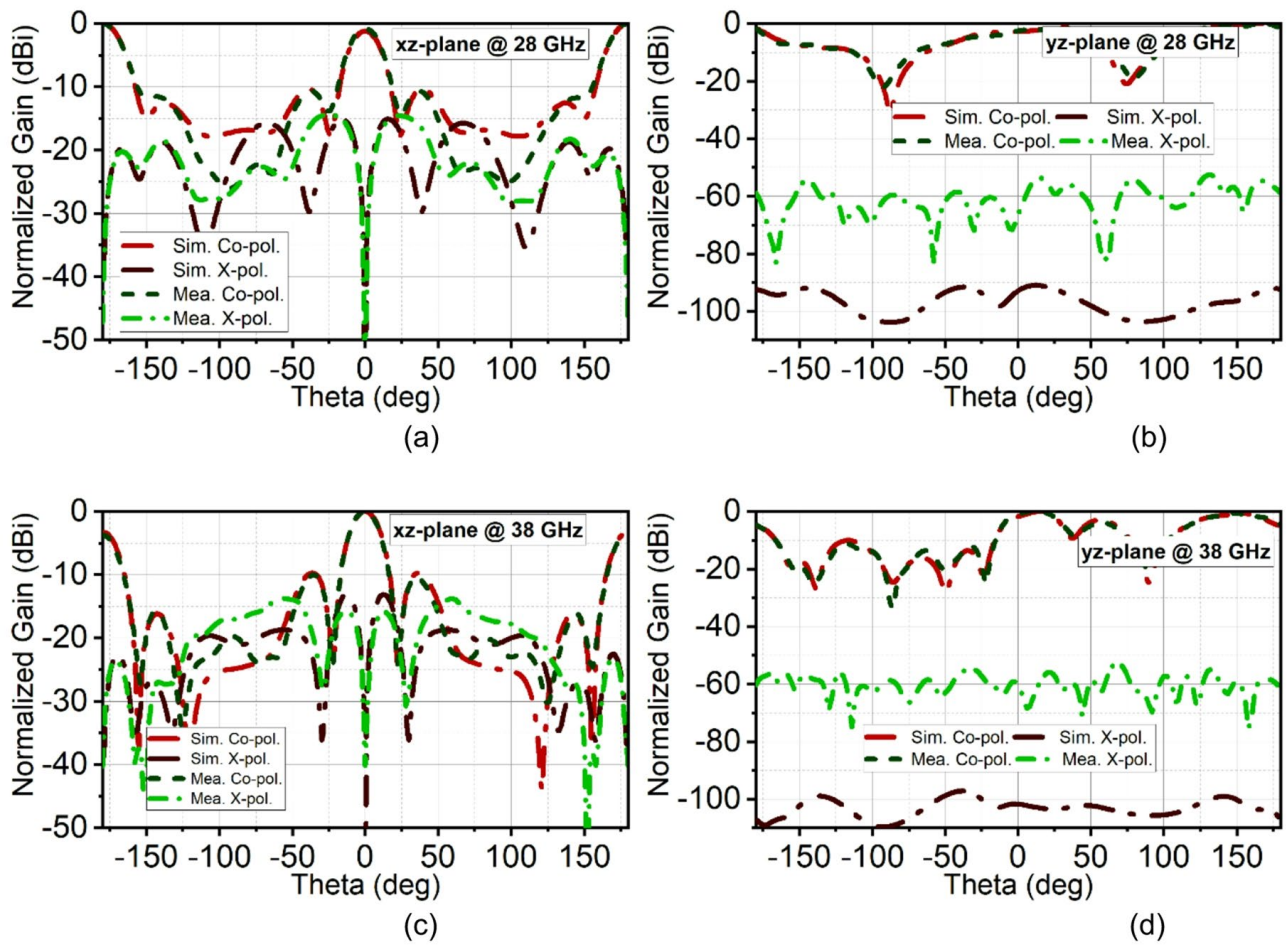
Simulated and measured radiation pattern of the proposed array antenna. (**a**) – (**b**) At 28 GHz, and (**c**)-(**d**) at 38 GHz.

**Fig. 17:**
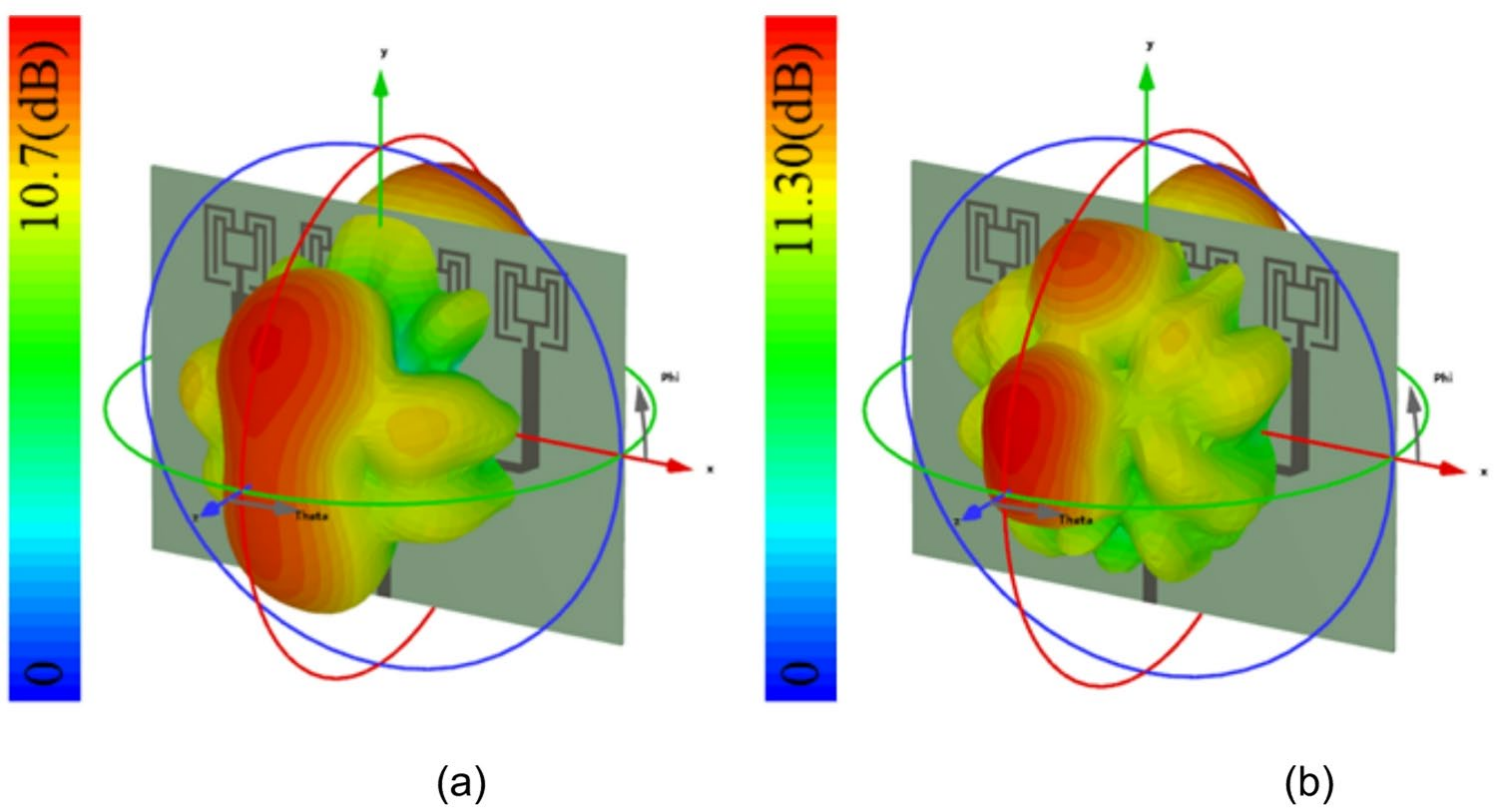
3D Far-field plots (**a**) 28 GHz and (**b**) 38 GHz.

The simulated and measured reflection coefficients |S11| are in good agreement. Simulated bandwidth ranges are 26.6–29.9 GHz and 34.1–39.1 GHz, whereas measured bandwidth ranges are 26.5–28.5 GHz and 33.75–39.12 GHz, as shown in Fig. 15. The array antenna has improved the directivity in the broadside direction forming narrow beam in the xz-plane (H-plane) and wide beam in the xz-plane (H-plane). The achieved maximum simulated and measured gains in the first band are 11 dBi and 10.7 dBi, respectively, and in the second band, it is 11.5 dBi. The simulated HPBW at 28 GHz is 17° with an SLL of −10 dB and X-polarization of −15 dB. The achieved measured HPBW is 18° with a slightly better SLL of −10.5 dB and X-polarization of −14.5 dB, as illustrated in Fig. 16(a). On the other hand, in the yz-plane, the simulated HPBW is 54° with a maximum X-polarization of −90 dB, whereas the measured HPBW is 49° with a maximum X-polarization of −55 dB, as depicted in Fig. 16(b). Similarly, at 38 GHz, the array antenna has achieved a narrow beam in the xz-plane and a wide beam in the yz-plane. The simulated HPBW in the xz-plane is 20° and measured is 19° with an SLL of −10 dB. The simulated and measured X-polarization is −13 dB and − 16 dB, respectively. In the other plane, that is in the yz-plane the simulated HPBW is 32.5° and measured is 34° with the X-polarization of −97 dB and − 59 dB, respectively, as depicted in Figs. 16(c) and (d). Figure 17 illustrates the 3D gain radiation patterns of the proposed antenna at 28 GHz and 38 GHz, highlighting its complex spatial behavior due to the partial ground plane configuration. At both frequencies, the antenna exhibits a non-uniform and asymmetrical radiation pattern with multiple lobes, confirming its dual-beam nature observed in the 2D far-field plots. The presence of strong lobes in opposite directions, aligned roughly along the x-axis is consistent with the dual-beam characteristics resulting from disrupted surface current flow caused by the truncated ground structure. The gain peaks at approximately 10.7 dB at 28 GHz and 11.3 dB at 38 GHz, indicating effective radiation performance despite the structural asymmetry.

To highlight the performance of proposed work, Table 2 shows the proposed design comparison with published literature. It can be seen that while most of literature focuses separately at 28 and 38 GHz band, the proposed work covers both resonances simultaneously with simple geometrical planar structure.

**Table 2:** Comparison of the proposed antenna with other existing designs.

						xz-plane			yz-plane		
Ref.	Dim. in mm^3^	Ant Type	Res. (GHz)	Bandwidth (GHz)	Gain (dBi)	HPBW	SLL (dB)	X-po. (dB)	HPBW	SLL (dB)	X-po. (dB)
^15^	24 × 18.85 × 0.254	Planar	28	26.5–29.45.5.45	9.1	22.7°	−1.4	< −9	36.2°	−1.7	< −18
^16^	--	SR + SIW Cavity	28/38	26.2–29.2/37–39	12.7/14.9	10°	−12	< −20	62°	-	< −20
^17^	$\:14\times\:32\times\:0.762$	Dual-loop	28/38	25.5–29.8/3.27–40.27	13/12	14°	−10	< −20	16°	−14	< −20
^18^	24 × 18.5 × 0.254	Planar	28	25.5–30	10.7	24°	−11	<−10.5	13.2°	NA	NA
^19^*	$\:37\times\:40\times\:2.1$	Planar Patch	27.5	26.4–28.3	9.8	40° (LHCP)	−10	< −12 (RHCP)	40° (RHCP)	−10	<−12 (LHCP)
^20^	$\:26\times\:6\times\:1.57$	Planar Patch + Series Feed	28	28–29.1.1	9.19	80°	−7	NA	18°	−2.5*	NA
^21^	$\:40\times\:40\times\:0.254$	MIMO Array + Corporate feed	30.5	30.4–31.5	9.8	40° (LHCP)	−8	−16	NA	NA	NA
^22^	--	Planar + Hybrid feed	24	23.7–24.4	14	16°	−14	NA	65°	-	NA
^23^	$\:32\times\:10\times\:0.254$	Non-uniform Dipole + Series feed + MTM	24	23–46	8.5–11.4	60°/30°	−12/−10	NA	55°/50°	−7/−10	NA
Prop.	$\:19.75\times\:26\times\:0.254$	Planar Patch + Corporate Feed	28/38	26.5–28.5/33.75–39.12	10.7/11.5	18°/19°	−10/−10	< −14.5/−16	49°/34°	-	< −55/−59

The^19^* is the design with CP characteristics.

## Conclusion

In this work, a compact dual key-shaped mmWave antenna for 28/38 GHz applications was designed and analyzed using the Theory of Characteristic Modes. The antenna, fabricated on a 0.254 mm substrate with dimensions of 10 × 12 mm², achieved fractional bandwidths of 12.5% and 21.05%, with Modes 1–4 dominating the 28 GHz resonance and Modes 2, 3, 4, 6, and 10 shaping the 38 GHz response. To improve gain and practical usability, the design was extended into a four-element linear array measuring 19.75 × 26 × 0.254 mm³, providing peak gains of 10.7 dBi at 28 GHz and 11.3 dBi at 38 GHz while maintaining more than 75% total efficiency. A fabricated prototype was tested using in-house facilities, and the measured performance closely matched the simulated results, confirming the effectiveness of the proposed approach. Future work may explore larger array configurations and MIMO implementations based on this design to meet diverse user requirements and support advanced 5G and beyond-5G communication systems.

## Data Availability

All data generated or analyzed during this study is included in this article.
